# Perceptions of Victimhood and Entrepreneurial Tendencies

**DOI:** 10.3389/fpsyg.2022.797787

**Published:** 2022-02-14

**Authors:** Yossi Maaravi, Boaz Hameiri, Tamar Gur

**Affiliations:** ^1^The Adelson School of Entrepreneurship, Reichman University, Herzliya, Israel; ^2^The Evens Program in Conflict Resolution and Mediation, Gershon H. Gordon Faculty of Social Sciences, Tel Aviv University, Tel Aviv, Israel; ^3^Department of Psychology, The Hebrew University of Jerusalem, Jerusalem, Israel; ^4^Baruch Ivcher School of Psychology, Reichman University, Herzliya, Israel

**Keywords:** victimhood, entrepreneurship, entrepreneurial personality, entrepreneurial tendencies, entrepreneurial intentions, self-efficacy

## Abstract

There is a growing scientific interest around entrepreneurship. One central line of research examines how different personality traits and characteristics such as creativity or resilience relate to entrepreneurial intentions and behavior. In the current research, we add to this literature by focusing on trait victimhood, a trait that entrepreneurship research has overlooked and may be relevant to understanding entrepreneurial tendencies. In two studies in Israel among a sample of entrepreneurship students (Study 1) and a sample representing the general public (Study 2), we show that trait victimhood is negatively related to entrepreneurial personality (Study 1) and behavior (Study 2). Moreover, Study 2 suggests that a strong sense of self-efficacy may buffer against trait victimhood’s adverse effects on behavioral entrepreneurship.

## Introduction

In the 21st century, the entrepreneurial approach is becoming central for individuals, organizations, and even nations (Kuratko, 2009; Jackson, 2011; Mozaffary, 2020). Hence, there is growing scientific research in entrepreneurship, with one line being entrepreneurial personality (Brandstätter, 2011; Obschonka et al., 2019). Entrepreneurial personality was recently characterized by: self-efficacy, autonomy, innovativeness, internal locus of control, achievement motivation, optimism, stress tolerance, and risk-taking (Cuesta et al., 2018; Gielnik et al., 2020), highlighting the role of internal locus of control and self-efficacy beliefs processes (Lüthje and Franke, 2003; Naushad and Malik, 2018). In the current research, we aim to contribute to this literature by exploring the relationship between entrepreneurship and trait victimhood (Gabay et al., 2020a,b), which was overlooked in this context and may be highly relevant to understanding entrepreneurial tendencies.

Trait victimhood is defined as *“an ongoing feeling that the self is a victim*… *generalized across many relationships, such that victimization becomes a central part of the individual’s identity.”* (Gabay et al., 2020a, p. 361). It fundamentally affects emotions, cognitions, and behaviors (Gabay et al., 2020b). We argue that trait victimhood is particularly conducive for the research on entrepreneurship because it provides a parsimonious conceptualization and measure, which is theoretically associated with several factors of entrepreneurial intent and behavior (Lüthje and Franke, 2003). Specifically, we argue that trait victimhood may be associated with an external locus of control (Gabay et al., 2020b) and higher perceived barriers. This is because people high in victimhood are more likely to be hyper-sensitive and expect hurtful behaviors and untrustworthiness by malevolent others in their daily lives (Gollwitzer et al., 2013).

**FIGURE 1:**
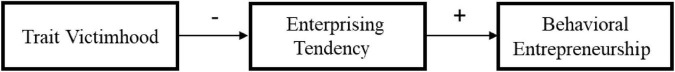
Conceptual model for trait victimhood and entrepreneurship.

Thus, in the current research, we explore the potential association between entrepreneurial tendencies and trait victimhood. Despite this research’s exploratory nature, we reasoned that trait victimhood would be negatively associated with entrepreneurship. For the conceptual model noting the researchers’ expectations see Figure 1. We examined the association between trait victimhood and entrepreneurship in two studies. These studies were conducted among a sample of entrepreneurship undergraduate students in Israel (Study 1) and an online sample drawn from the general population of adults in Israel (Study 2).

## Study 1

### Method

#### Participants and Procedure

We sampled 208 entrepreneurship undergraduate students at a private university in Israel (40.9% women; *M*_age_ = 23.74, SD = 2.19) that received course credits in exchange for their participation. Participants responded to an online questionnaire with the measures detailed below, following which they reported their socio-demographic information. Unless indicated otherwise, all items in both studies were rated on a 7-point Likert-type scale ranging from (1) “strongly disagree” to (7) “strongly agree” (for complete information, see Supplementary Material).

#### Measures

*Trait victimhood* was assessed with an abridged 9-item version of the tendency for interpersonal victimhood scale (Gabay et al., 2020b; e.g., “It is important to me that people who hurt me acknowledge that an injustice has been done to me”; α = 0.73).

*Enterprising Tendency* was assessed with the 54-item GET2 test (Caird, 2006) on a dichotomous scale anchored at (0) “disagree” and (1) “agree” (e.g., “At work, I often take over projects and steer them my way without worrying about what other people think”), which were summed to obtain the GET2 score (α = 0.65).

*Behavioral Entrepreneurship* was assessed by asking participants to report whether they founded at least one business initiative in the past (29.8% of the sample reported that they did).

### Results and Discussion

For variable means, SDs and correlations see Table 1. Looking at the simple bivariate correlations, trait victimhood was significantly negatively correlated with enterprising tendency, but not with behavioral entrepreneurship. These associations did not meaningfully change when we controlled for participants’ age and gender.

**TABLE 1:** Means, SDs, and correlations of study variables (Studies 1 and 2).

	Mean (SD)	1	2	3	4	5
**Study 1**						
Trait victimhood	3.53(0.91)	–				
Enterprising tendency	37.92(4.84)	−0.22**	–			
Behavioral entrepreneurship (0 = N, 1 = Y)	–	–0.10	0.29**	–		
Age	23.74(2.19)	0.02	–0.03	0.12	–	
Gender (1 = M, 2 = F)	–	–0.06	–0.02	–0.07	−0.24**	–
**Study 2**						
Trait victimhood	4.54(0.99)	–				
Global self-efficacy	5.33(0.85)	0.14**	–			
Behavioral entrepreneurship (0 = N, 1 = Y)	–	–0.02	0.17**	–		
Age	41.71(16.02)	0.003	0.01	–0.08	–	
Gender (1 = M, 2 = F)	–	0.09	0.04	–0.10	0.02	–

** p < 0.01.

While a moderate association was found between enterprising tendency and behavioral entrepreneurship, trait victimhood was unexpectedly not associated with behavioral entrepreneurship. Possibly we have not been able to detect this effect due to the current study’s limitations. Specifically, the sample in Study 1 was comprised of entrepreneurship students, which does not represent the general public. Indeed, it yielded a remarkably lower trait victimhood mean score (*M* = 3.53, SD = 0.91) compared to other studies conducted in Israel (Gabay et al., 2020b) and in the United States (Hameiri et al., 2020), where means were around or above the scale’s midpoint (4).

Another potential explanation for the lack of the above association might be the result of a moderating agent. Previous work has established the pivotal role of self-efficacy, i.e., “beliefs in one’s capabilities to mobilize the motivation, cognitive resources, and courses of action needed to meet given situational demands” (Wood and Bandura, 1989, p. 408), plays in entrepreneurial intent and behavior (Singh, 1978; Naushad and Malik, 2018). Thus, while individuals might have an enterprising tendency, actual engagement in entrepreneurship also depends on their beliefs of pursuing and succeeding despite possible difficulties (Chen et al., 1998).

**FIGURE 2:**
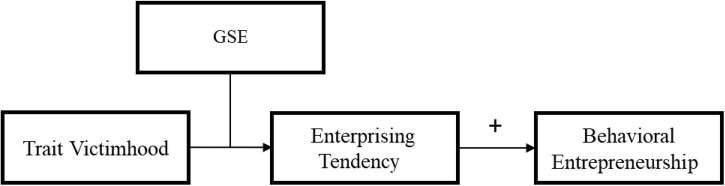
The revised conceptual model for trait victimhood and entrepreneurship.

In Study 2, we addressed the methodological limitation by sampling participants representing the general population in Israel. Furthermore, we introduced a measure of global self-efficacy (GSE) to examine whether it moderated the relationship between trait victimhood and (self-reported) behavioral entrepreneurship. Self-efficacy measures during entrepreneurship training were recently associated with business ownership (Gielnik et al., 2020). We reasoned that those who think they can get their initiatives off the ground would generally show high behavioral entrepreneurship levels, irrespective of their level of trait victimhood. In contrast, those who believe that they are less efficacious would be more affected by their tendency for victimhood. The model was revised accordingly to entail GSE and the components of the initial model (see Figure 2).

## Study 2

### Method

#### Participants and Procedure

We sampled 354 Jewish-Israelis (48.6% women; *M*_age_ = 41.71, SD = 16.02) to participate in an online study *via* an Israeli survey company (Midgam Project). Participants responded to measures detailed below and provided some socio-demographic information.^1^

#### Measures

*Trait victimhood* (α = 0.82) was measured with the same items as in Study 1.

*Global Self-Efficacy* was measured with the 10-item GSE scale (Schwarzer et al., 1999; e.g., “I can always manage to solve difficult problems if I try hard enough”; α = 0.91).

*Behavioral Entrepreneurship* was measured with the same item as Study 1. We added three more items asking participants to indicate whether they are currently self-employed, are planning to become self-employed in the foreseeable future, and whether they founded any social initiatives. Participants’ score was computed such that those who responded “yes” on *any* of these four items received 1 (52.3% of the sample), and those who answered “no” on *all* items received 0.

### Results and Discussion

**FIGURE 3:**
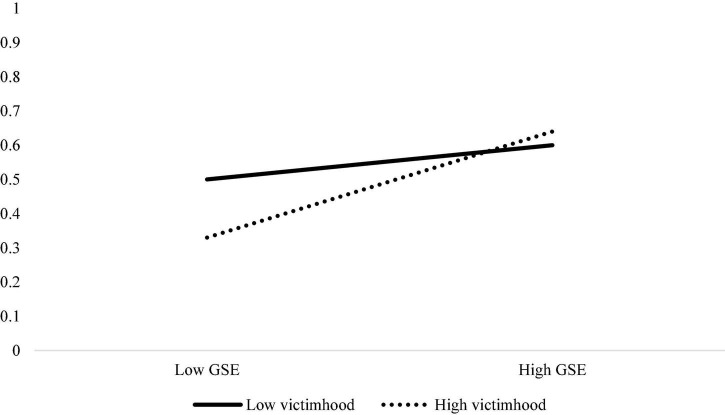
Probability to engage in behavioral entrepreneurship as a function of the interaction between trait victimhood and global self-efficacy (Study 2).

For means, SDs and correlations, see Table 1. Looking at the simple bivariate correlations, trait victimhood was not correlated with behavioral entrepreneurship. Interestingly, trait victimhood was positively correlated with GSE. Predictably, GSE was positively correlated with behavioral entrepreneurship. We tested the trait victimhood × GSE interaction on behavioral entrepreneurship, using Hayes’s (2018) PROCESS (Model 1) bootstrapping command with 5,000 iterations. Similar to Study 1, trait victimhood did not significantly predict behavioral entrepreneurship [*b* = −0.15, *se* = 0.12, *p* = 0.201, 95% Confidence Interval (CI) = (−0.38, 0.08)]; while GSE did [*b* = 0.49, *se* = 0.14, *p* < 0.001, 95% CI = (0.22, 0.76)]. More importantly, the analysis yielded a significant interaction [*b* = 0.29, *se* = 0.13, *p* = 0.027, 95% CI = (0.03, 0.55); see Figure 3]. Conditional effects revealed that trait victimhood was significantly negatively associated with behavioral entrepreneurship among the low-GSE participants [*b* = −0.40, *se* = 0.18, *p* = 0.029, 95% CI = (−0.76, −0.04)], but not among the high-GSE participants [*b* = 0.10, *se* = 0.14, *p* = 0.471, 95% CI = (−0.17, 0.38)]. These effects hold when controlling for age and gender.

Finally, we included several measures to replicate Lüthje and Franke’s (2003) entrepreneurial intent model. While they were not reliable (αs < 0.62), and were omitted from the final analysis, correlations between trait victimhood and individual items from these measures provide additional tentative corroboration for our reasoning. Specifically, trait victimhood was positively correlated with both locus of control items (e.g., “I often feel that that’s how things are and there’s nothing I can do about it”; *r*s > 0.21, *p*s < 0.001), and with all three perceived barriers items (e.g., “State laws are adverse to running a company”; *r*s > 0.15, *p*s < 0.004).

## General Discussion

It seems that there is consensus around the “entrepreneurial imperative of the twenty-first century” (Kuratko, 2009), which also led to flourishing scientific research around its different aspects—including a specific focus on the personality traits (or lack thereof) that characterize entrepreneurs. The present research explored the association between trait victimhood and entrepreneurship. While potentially a parsimonious conceptual and empirical construct in entrepreneurship, trait victimhood was overlooked in this context. Indeed, in two studies among either a sample of Israeli entrepreneurship students or a sample representing the Jewish-Israeli general public, trait victimhood is inversely related to entrepreneurial personality and behavior. Furthermore, having a strong sense of self-efficacy buffers against trait victimhood’s adverse effects on behavioral entrepreneurship.

### Theoretical and Practical Implications

This research has several important theoretical and practical implications. First, the research on trait victimhood has explored how it shapes people’s emotions, cognition, and behavior. Two recent examples are people’s willingness to forgive those who hurt them and seek revenge (Gabay et al., 2020b) and adherence to health guidelines in the COVID-19 pandemic (Maaravi et al., 2020a). To the best of our knowledge, the current study is the first to show that trait victimhood might also be associated with people’s career choices or their ability to succeed as entrepreneurs. Correspondingly, levels of trait victimhood were significantly lower among the sample of entrepreneurship undergraduate students in Study 1 (*M* = 3.53, SD = 0.91) than in the sample that resembled the Jewish population in Israel in Study 2 (*M* = 4.54, SD = 0.99; *p* < 0.001) and compared to previous studies conducted in Israel and the United States (Gabay et al., 2020b; Hameiri et al., 2020; Maaravi et al., 2020a).

Furthermore, the current research adds to our understanding of the psychology of entrepreneurship. As part of the nature-versus-nurture debate of entrepreneurship, some scholars argue that entrepreneurship can be taught or at the very least encouraged (Maaravi et al., 2020b). The current research suggests that high trait victimhood individuals are less likely to benefit from such endeavors. However, one way to circumvent this potential barrier is by bolstering aspiring entrepreneurs’ self-efficacy (e.g., Naushad and Malik, 2018).

These last implications bear theoretical and significant practical importance since self-efficacy is highly affected by environmental cues. It can be induced *via* peer modeling (exposure to peers who successfully ventured) and persuasion (speaking to the person of their competence; Dale and Barry, 2007). However, all these intriguing avenues should be thoroughly examined in future research.

### Limitations and Future Research

Beyond the below limitations, there are no specific “Limitations on Generality” (Simons et al., 2017), such as specific populations or specific experimental materials. However, it should be noted that the current research is correlational. Thus, we cannot decisively conclude that trait victimhood or GSE had a causal effect on entrepreneurial behavior. Future research should attempt to establish causality by, for example, priming a sense of victimhood. However, our hypothesized independent variables were personality traits, which should precede and predict behavior. Another limitation is that, albeit each study utilized a different sample, both studies were conducted with Jewish-Israeli participants. Thus, it is important to replicate these findings in other contexts.

## Conclusion

To summarize, we argue that trait victimhood offers a simple yet powerful measure of individual differences when facing challenges and hardships, which are the reality of entrepreneurship and the creative process. We also present a likely moderator that may bolster people against the effects of trait victimhood.

## Data Availability Statement

The data that support the findings of this study are openly available at https://osf.io/7e69t/?view_only=91d5c2db4b1346cd8db2a549fbf42bc6.

## Ethics Statement

The studies involving human participants were reviewed and approved by the Adelson School of Entrepreneurship Ethics Committee as part of the Institutional Review Board (IRB), IDC Herzliya. The patients/participants provided their written informed consent to participate in this study.

## Author Contributions

YM: study conceptualization, project management, data collection, and report writing. BH: study conceptualization, data collection, data preparation, data analysis, and report writing. TG: data collection, data preparation, data analysis, and report writing. All authors contributed to the article and approved the submitted version.

## Conflict of Interest

The authors declare that the research was conducted in the absence of any commercial or financial relationships that could be construed as a potential conflict of interest.

## Publisher’s Note

All claims expressed in this article are solely those of the authors and do not necessarily represent those of their affiliated organizations, or those of the publisher, the editors and the reviewers. Any product that may be evaluated in this article, or claim that may be made by its manufacturer, is not guaranteed or endorsed by the publisher.

## Footnotes

1 This was part of a larger study that included other variables that were published in a separate paper (for further details see Supplementary Material).
