# Supplementary material for: Perceptions of Victimhood and Entrepreneurial Tendencies
Source: Front Psychol. 2022 Feb 14;13:797787. doi: 10.3389/fpsyg.2022.797787 (PMC8882629; doi:10.3389/fpsyg.2022.797787)
Supplement: Supplementary file 3 [file Data_Sheet_3.pdf]

Perceptions of victimhood and entrepreneurial tendencies- Supplementary materials: Study 2

This was part of a larger study that included other variables, some of which were published in a separate paper (Maaravi, Hameiri & Gur, 2020), and some will be published elsewhere.

<u>The examined variables were:</u>

- Trait victimhood
- GSE
- Behavioral Entrepreneurship
- NFS (assess mental rigidity)
- Fear of the coronavirus (COVID fear)
- Adherence to Israel's health department regulations regarding protection from COVID
- A modified version of Lüthje and Franke's (2003) entrepreneurial intent

<u>Lüthje and Franke's (2003) entrepreneurial intent model replication.</u>

Risk taking propensity. A 3 items measure (α = .53).

Locus of control. A 2 items measure (*r* = .31).

Perceived barriers. A 3 items measure (α = .41).

Perceived support factors. A 3 items measure (α = .45).

Attitude towards entrepreneurship. A 3 items measure (α = .59).

Maaravi, Y., Hameiri, B., and Gur, T. (2020). Fighting coronavirus one personality at a time: Need for structure, trait victimhood, and adherence to COVID-19 health guidelines. *Frontiers in Psychology*, 11.

Lüthje, C., and Franke, N. (2003). The 'making' of an entrepreneur: Testing a model of entrepreneurial intent among engineering students at MIT. *R&D Management, 33,* 135–147.
